# Chlorpyrifos oxon promotes tubulin aggregation via isopeptide cross-linking between diethoxyphospho-Lys and Glu or Asp: Implications for neurotoxicity

**DOI:** 10.1074/jbc.RA118.004172

**Published:** 2018-07-13

**Authors:** Lawrence M. Schopfer, Oksana Lockridge

**Affiliations:** From the Eppley Institute, University of Nebraska Medical Center, Omaha, Nebraska 68198-5900

**Keywords:** tubulin, neurotoxin, protein misfolding, mass spectrometry (MS), Western blot, crosslink, isopeptide, neurotoxicity, organophosphate, protein aggregation

## Abstract

Exposure to organophosphorus toxicants (OP) can have chronic adverse effects that are not explained by inhibition of acetylcholinesterase, the cause of acute OP toxicity. We therefore hypothesized that OP-induced chronic illness is initiated by the formation of organophosphorus adducts on lysine residues in proteins, followed by protein cross-linking and aggregation. Here, Western blots revealed that exposure to the OP chlorpyrifos oxon converted porcine tubulin from its original 55-kDa mass to high-molecular-weight aggregates. Liquid chromatography–tandem MS analysis of trypsin-digested samples identified several diethoxyphospho-lysine residues in the OP-treated tubulin. Using a search approach based on the Batch Tag program, we identified cross-linked peptides and found that these chemically activated lysines reacted with acidic amino acid residues creating γ-glutamyl-ϵ-lysine or aspartyl-ϵ-lysine isopeptide bonds between β- and α-tubulin. Of note, these cross-linked tubulin molecules accounted for the high-molecular-weight aggregates. To the best of our knowledge, this is the first report indicating that chlorpyrifos oxon–exposed tubulin protein forms intermolecular cross-links with other tubulin molecules, resulting in high-molecular-weight protein aggregates. It is tempting to speculate that chronic illness from OP exposure may be explained by a mechanism that starts with OP adduct formation on protein lysines followed by protein cross-linking. We further speculate that OP-modified or cross-linked tubulin can impair axonal transport, reduce neuron connections, and result in neurotoxicity.

## Introduction

The accepted mechanism for acute toxicity from exposure to organophosphorus toxicants is inhibition of acetylcholinesterase (AChE)^3^ activity (1). AChE activity returns to normal levels within 1 month, but chronic illness can persist for a lifetime. Persistent conditions that develop after acute intoxication can be divided into three categories. The first is organophosphate-induced delayed neuropathy, which is initiated by inhibition and aging of neuropathy target esterase (2). Symptoms include muscular incoordination, numbness, tingling, fatigue, severe muscular weakness, and paralysis of the lower limbs (3). These are associated with degeneration of axons, along with demyelination. Only a subset of organophosphorus toxicants (OP), including leptophos, diisopropylfluorophosphate, mipafox, and tri-*o*-cresyl phosphate cause this condition (4). The second category has been described as a post-traumatic stress disorder and is characterized by decreases in intellectual functioning, academic skills, and flexibility of thinking. In addition there is memory loss and loss of simple motor skills. Subjects also develop problems with depression, irritability, confusion, and social withdrawal (4). The physiological basis for these persistent conditions remains unknown.

A third source of persistent symptomology comes from repeated exposure to low levels of organophosphorus pesticides, levels that are too low to observe acute symptoms of inhibition of AChE activity. This can lead to chronic illness in adults manifested as deficits in memory, attention, reaction time, and fine motor skills, plus emotional difficulties (5–8). Children exposed *in utero* to the OP chlorpyrifos can exhibit developmental abnormalities (9–11). Studies in cultured cells and laboratory rodents exposed to low levels of OP have provided evidence that multiple pathways are potentially affected by low doses of chlorpyrifos (CPS) and its active metabolite, chlorpyrifos oxon (CPO). These include altered receptor levels, adenylyl cyclase activity, and signal transduction activity, increased phosphorylation of Ca^2+^/cAMP-response element-binding protein, and impaired axonal transport (12–14). These studies did not identify molecular targets for CPS or CPO and did not propose a mechanism for neurotoxicity.

Covalent modification of noncholinesterase proteins may provide a rational basis for these symptomatic observations. Before 2007, it was generally accepted that OP reacted primarily with the active site serine of esterases and proteases. A limited number of reports had appeared describing reactions of OP with tyrosine in proteins such as human serum albumin (15), bromelain (16), papain (17), and hen egg white lysozyme (18). Beginning in 2007 we confirmed and amplified these observations using MS. We found OP adducts on both tyrosine and lysine in tubulin, albumin, casein, aprotinin, and keratin (19–23). The OP in these studies included soman, sarin, chlorpyrifos oxon, dichlorvos, diisopropylfluorophosphate, and a biotinylated OP probe, FP-biotin. Other laboratories have reported tyrosine adducts on albumin and other proteins (24–30).

Schmidt *et al.* (31) demonstrated that phosphonylation of lysine residues in ubiquitin by VX (*O*-ethyl methyl S-2-(diisopropylaminoethyl)phosphonothioate)) induced intramolecular cyclization through the formation of isopeptide bonds with nearby glutamate residues in the same protein subunit. The Schmidt *et al.* (31) report led us to the idea that CPO could induce cross-linking between subunits of tubulin. We chose to work with tubulin because tubulin is the major constituent of microtubules in the brain, microtubules being critical for cell morphology and cell division, and because of our previous results that showed modification of microtubule structure in mice treated with CPS (32). We searched for intermolecular cross-links between subunits because such cross-links would be expected to cause protein aggregation. We reasoned that aggregation of tubulin would disrupt microtubule structure, and would be a likely explanation for the altered microtubule structure we observed in mouse brain.

In the present report we treated porcine tubulin with CPO and examined the products for modifications by SDS-PAGE, Western blotting, and MS. SDS-PAGE and Western blotting revealed that treatment with CPO led to extensive tubulin aggregation. Mass spectrometry identified diethoxyphospho-lysine on both α- and β-tubulin. In addition, it showed that lysine residues susceptible to CPO-labeling were involved in isopeptide cross-links with glutamate and aspartate. Cross-links were found between residues on different peptides within the same form of tubulin and between residues on different forms of tubulin.

## Results

### Chlorpyrifos oxon induced aggregation of tubulin

**Figure 1.**
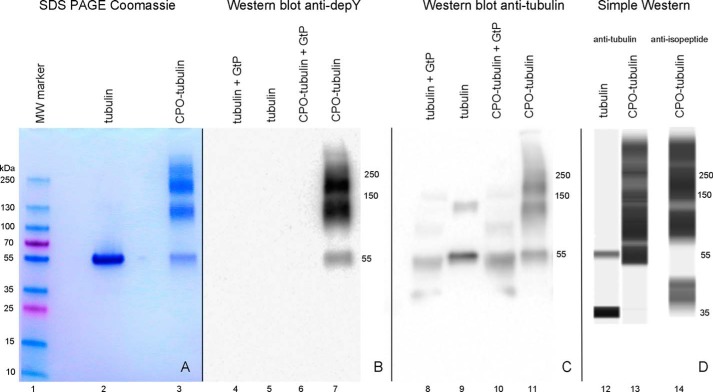
**SDS-PAGE stained with Coomassie Blue (*lanes 1–3*), Western blotting on PVDF membrane probed with anti-diethoxyphosphotyrosine antibody depY (*lanes 4–7*), Western blotting probed with anti-tubulin antibody (*lanes 8–11*), and a capillary electrophoresis Simple Western image (*lanes 12–14*) probed with anti-tubulin antibody (*lanes 12* and *13*) or anti-isopeptide antibody (*lane 14*).** All samples were reduced with DTT prior to electrophoresis. The band at 35 kDa for untreated tubulin (*lane 12*) was an artifact of the Simple Western procedure.

Fig. 1 shows that treatment of tubulin with 1.5 mm CPO leads to protein aggregation. Untreated control tubulin has a band at 55 kDa (*lanes 2* and *9*). Extensive aggregation can be seen for CPO-treated tubulin on the Coomassie-stained SDS-PAGE (*lane 3*), on the anti-diethoxyphosphotyrosine Western blotting (*lane 7*), on the anti-tubulin Western blotting (*lane 11*), and on the Simple Western using either the anti-tubulin antibody (*lane 13*) or the anti-isopeptide antibody (*lane 14*). A weak band at 130 kDa in *lane 9* for untreated tubulin on the anti-tubulin Western blotting had no isopeptide cross-links. Treatment of tubulin or CPO-tubulin with γ-glutamyltranspeptidase reduced the size of the aggregate bands (*lane 10*), suggesting that isopeptide bonds are involved in aggregate formation. Taken together, these results argue that CPO promotes isopeptide bond cross-linking of tubulin monomers to make multimers.

The anti-tubulin Western blotting reveals that after γ-glutamyltranspeptidase treatment the tubulin monomer (*lane 8*) is slightly smaller than untreated tubulin (*lane 9*). This suggests that some proteolysis may have taken place. We found that the commercial γ-glutamyltranspeptidase preparation was highly contaminated with other proteins.

**Figure 2.**
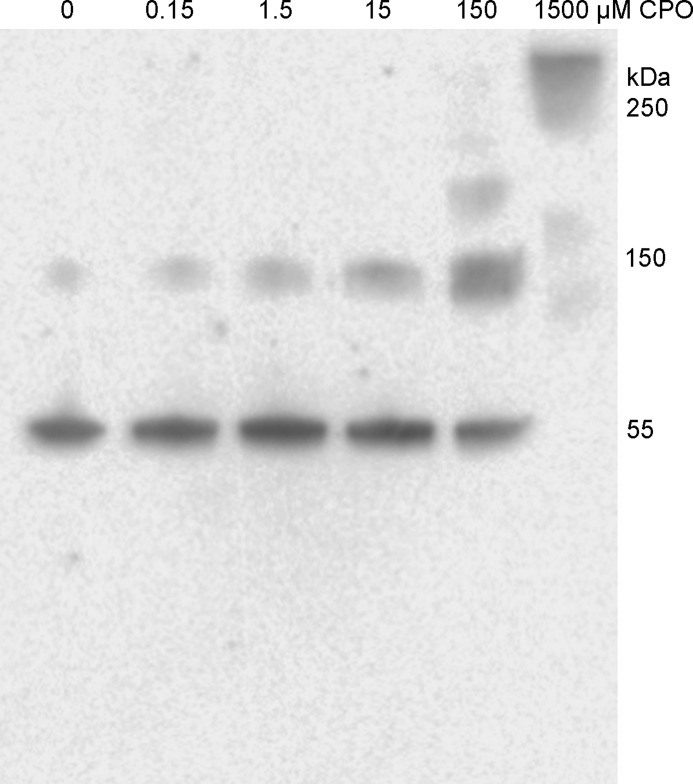
**Western blotting on PVDF membrane probed with anti-tubulin antibody.** Tubulin (0.25 mg/ml) was treated with 0 to 1500 μm CPO. Each lane of the SDS gel was loaded with 1 μg of tubulin after the sample was reduced with DTT and denatured in the presence of SDS in a boiling water bath.

To clarify whether the high molecular species detected in the gels arose from multiple or single cross-linking events that are destabilized enough to form large aggregates we treated 0.25 mg/ml of tubulin with different concentrations of CPO (0, 0.15, 1.5, 15, 150, and 1500 μm). Fig. 2 shows that concentrations of 0.15, 1.5, and 15 μm CPO resulted in increasing amounts of trimer at 130 kDa. Treatment with 150 μm CPO caused higher molecular weight (200 to 300 kDa) aggregates to appear. At 1500 μm all of the tubulin was present as high-molecular-weight aggregates with no monomer and very little trimer (130 kDa). The change in distribution suggests that cross-linking initially forms trimers, but at sufficiently high concentrations of CPO multiple cross-links develop.

### Organophosphorylated tubulin peptides

Trypsin-digested control tubulin and CPO-treated tubulin samples were separated by ultra HPLC prior to electrospray ionization MS on a Triple TOF 6600 mass spectrometer. The mass spectral data were searched against the uniprot_sprotJan2015.fasta database using the Paragon algorithm from Protein Pilot to identify the organophosphorylated peptides. Candidates were manually sequenced to confirm the Protein Pilot assignment and to identify the labeled amino acids.

We found no diethylphosphate-labeled peptides in control tubulin. In contrast CPO-treated tubulin contained the diethylphosphate-labeled peptides listed in Table 1.

**Table 1:** **Diethoxyphosphorylated lysine and tyrosine in CPO-treated tubulin** Only peptides with Protein Pilot Confidence values of 95% or greater are listed.

Peptide*^a^*	Tubulin UniProt	Labeled AA	Residue	MS/MS	Ratio*^b^* label/total
QLFHPEQLITGK*EDAANNYAR	P02550 α1A	Lysine	96	Yes	0.099
GHYTIGK*EIIDLVLDR	P02550	Lysine	112	Yes	0.34
LSVDYGK*K	P02550	Lysine	163	Yes	0.28
LSVDYGK*K*SK	P02550	Lysine	163, 164	Yes	–
HGK*YMACCLLYR	P02550	Lysine	311	Yes	0.64
DVNAAIATIK*TK	P02550	Lysine	336	Yes	0.052
DVNAAIATIK*TK*R	P02550	Lysine	336, 338	Yes	–
VGINYEPPTVVPGGDLAK*VQR	P02550	Lysine	370	No	0.35
LDHK*FDLMYAK	P02550	Lysine	394	Yes	0.33
FDLMYAK*R	P02550	Lysine	401	Yes	0.31
IHFPLATYAPVISAEK*AYH	Q2XVP4 α1B	Lysine	280	Yes	0.083
K*VGINYQPPTVVPGGDLAK	Q2XVP4	Lysine	352	Yes	0.043
ISVYYNEATGGK*YVPR	Q767L7 β	Lysine	58	No	0.011
K*GHYTEGAELVDSVLDVVR	Q767L7	Lysine	103	No	0.19
QLTHSLGGGTGSGMGTLLISK*IR	Q767L7	Lysine	154	Yes	0.29
IMNTFSVVPSPK*	Q767L7	Lysine	174	Yes	0.012
TLK*LTTPTYGDLNHLVSATM	Q767L7	Lysine	216	Yes	0.50
K*LAVNMVPFPR	Q767L7	Lysine	252	Yes	0.18
MSMK*EVDEQMLNVQNK	Q767L7	Lysine	324	No	0.18
EVDEQMLNVQNK*NSSYFVEWIPNNVK	Q767L7	Lysine	336	No	0.011
GLK*MAVTFIGNSTAIQELFK	Q767L7	Lysine	362	Yes	0.50
STAIQELFK*R	Q767L7	Lysine	379	Yes	0.27
K*AFLH	Q767L7	Lysine	392	Yes	1.00
ALTVPELTQQMFDAK*NMMAACDPR	P02554 β	Lysine	297	No	0.0059
EDAANNY*AR	P02550 α1A	Tyrosine	103	Yes	0.35
LSVDY*GK	P02550	Tyrosine	161	Yes	0.41
LEFSIY*PAPQVSTAVVEPYNSILTTH	P02550	Tyrosine	172	No	0.14
LDIERPTY*TNLNR	P02550	Tyrosine	224	Yes	0.50
EFQTNLVPY*PR	P02550	Tyrosine	262	Yes	0.12
Y*APVISAEK	P02550	Tyrosine	272	Yes	0.33
Y*MACCLLYR	P02550	Tyrosine	312	Yes	0.34
VGINY*EPPTVVPGGDLAK	P02550	Tyrosine	357	No	0.29
AFVHWY*VGEGM	P02550	Tyrosine	408	Yes	0.18
ISVY*YNEATGGK	Q767L7 β	Tyrosine	50	Yes	0.079
ISVYY*NEATGGK	Q767L7	Tyrosine	51	No	0.10
ISVYYNEATGGKY*VPR	Q767L7	Tyrosine	59	No	0.50
GHY*TEGAELVDSVLDVVR	Q767L7	Tyrosine	106	Yes	0.17
Y*LTVAAVFR	Q767L7	Tyrosine	310	Yes	0.34
NSSY*FVEWIPNNVK	Q767L7	Tyrosine	340	Yes	0.28
AFLHWY*TGEGMDEM	Q767L7	Tyrosine	398	Yes	0.57

*^a^* Peptides were from a tryptic digestion. Diethoxyphosphorylated residues are followed by an asterisk (*).

*^b^* The ratio is the number of times a peptide containing a diethoxyphosphorylated residue appears in the mass spectral data divided by the total number of times a peptide containing that residue appears. Peptides containing 2 labeled lysines are indicated by a dash in the last column.

Tubulin α1A (P02550) and α1B (Q2XVP4) each contain 19 lysines in homologous positions. Database searching with Protein Pilot found 14 of these lysines to be labeled by CPO. Twelve of the labeled peptides were identified with confidence values of 95% or greater. These are shown in Table 1. Peptide IHFPLATYAPVISAEKAYH is unique to α1B by virtue of the Ala to Ile substitution at position 1. The ratio of labeled to unlabeled peptides in this group ranged from 0.043 to 0.64. The sequences and labeled residues for 11 peptides were confirmed by manual sequencing of the mass spectral fragmentation spectra (Table 1). α1A- and α1B-tubulins also contain 19 tyrosines. Thirteen were found to be labeled by CPO, 9 with confidence values of 95% or greater (at ratios of 0.12 to 0.50), of which 7 were confirmed by manual sequencing.

Tubulin β (P02554 and Q767L7) contains 15 lysines. Thirteen lysines were found to be labeled by CPO, 12 with confidence values of 95% or greater (at ratios of 0.0059 to 1.00), of which 7 were confirmed by manual sequencing (Table 1). Peptide ALTVPELTQQ**M**FDAKNMMAACDPR is unique to β P02554 by virtue of the Val to Met substitution at position 11. β-Tubulins also contain 16 tyrosines. Ten tyrosines were found to be labeled by CPO, 7 with confidence values of 95% or greater (at ratios of 0.079 to 0.57), of which 5 were confirmed by manual sequencing. These observations clearly demonstrate that both lysine and tyrosine are labeled by CPO and that the efficiency of labeling varies considerably from residue to residue.

### Cross-linked tubulin peptides

We found no isopeptide cross-linked peptides in control tubulin. By contrast, CPO-treated tubulin contained the cross-linked peptides listed in Table 2. Mass spectra of the digests were obtained by electrospray ionization using a Triple TOF 6600 mass spectrometer. Cross-links were identified using the Batch Tag and Search Compare algorithms from ProteinProspector. Batch Tag searches were constrained to find isopeptide cross-links: (*a*) between lysine and glutamate, aspartate, glutamine, or asparagine; or (*b*) between lysine and any amino acid. Cross-linked peptide candidates reported by Search Compare were confirmed by manual sequencing. The same isopeptide bonds were found when Batch Tag was configured to identify cross-links between lysine and any amino acid, or between lysine and glutamate, aspartate, glutamine, or asparagine. Cross-links between α- and β-tubulin were observed. When both peptides in a cross-link were from β-tubulin or from α-tubulin, we could not determine whether the peptides came from the same or distinct molecules.

**Table 2:**
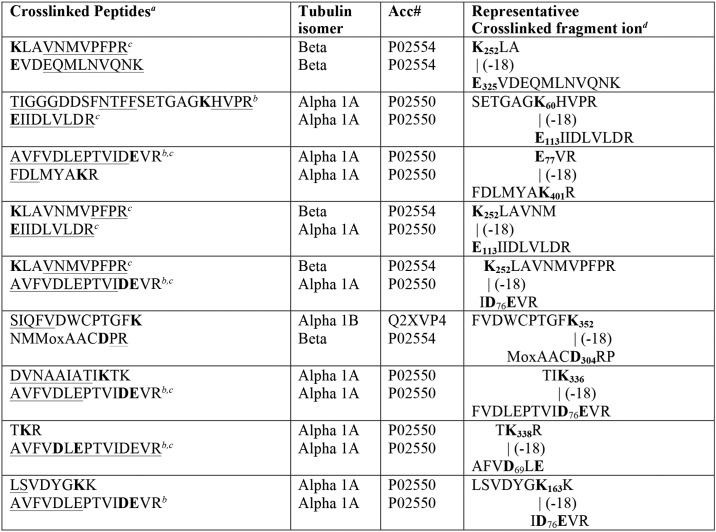
**Summary of cross-linked tubulin peptides**

*^a^* Sequences for the parts of each peptide that were observed in the MS/MS spectrum are underlined (singly-charged sequence and doubly-charged sequence). Residues involved in the crosslink are shown in **bold**. When there was a choice for labeled residue that could not be resolved, both candidate residues are shown in **bold**. Cross-linked lysines were diethoxyphosphorylated prior to forming the isopeptide bond.

*^b^* An acidic or basic residue near the cross-link promotes isopeptide bond formation.

*^c^* This peptide or a portion of this peptide is used in more than one crosslinked pair.

*^d^* A crosslinked fragment ion from the MS/MS spectrum is shown. Numbering indicates the position of the residue in the primary sequence. The numbering includes the leader sequence. The mass of the cross-linked peptides is the sum of the two peptides minus 18 Da for the isopeptide linkage.

The MS/MS fragmentation spectrum for each cross-linked peptide contained portions of sequence from each peptide in the pair (*underlined* in Table 2) plus masses consistent with cross-linked portions from both peptides (a representative fragment ion from the MS/MS spectrum for each cross-linked peptide is shown in Table 2). In most cases the cross-linked fragments appeared in a sequence of doubly-charged masses.

**Figure 3.**
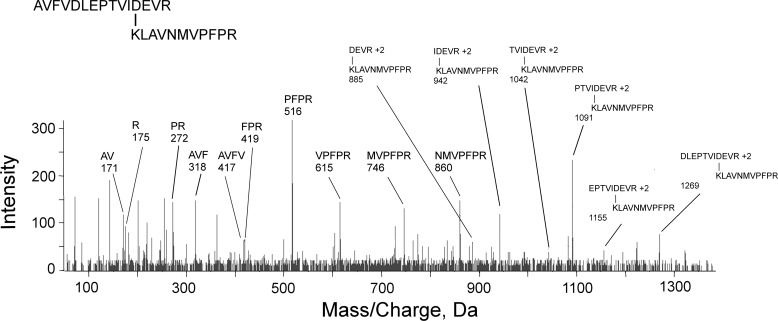
**A mass spectral fragmentation spectrum for the cross-linked peptides AVFVDLEPTVIDEVR from tubulin α1A (P02550) and KLAVNMVPFPR from tubulin β (P02554).** Parent ion 739.4 [M + 4H]^+4^ was not detected in the fragmentation spectrum. Unlabeled masses correspond to internal fragments, immonium ions, and *b*- or *y*-ions minus water and/or amine. The mass of cross-linked peptides is decreased by 18 Da due to loss of a water molecule upon formation of the isopeptide bond.

A representative MS/MS fragmentation spectrum for a cross-linked peptide is in Fig. 3. This spectrum is for the cross-linked peptides KLAVNMVPFPR (β, P02554) and AVFVDLEPTVIDEVR (α1A, P02550). It consists of three components: 1) the N-terminal *b*-series AVFV from AVFVDLEPTVIDEVR; 2) the C-terminal *y*-series NMVPFPR from KLAVNMVPFPR; and 3) a doubly-charged series for masses, which are too large to correspond to either peptide by itself. The doubly-charged series matches the sequence from AVFVDLEPTVIDEVR. Those masses decrease in the order DLEPTVI, which indicates that this is a *y*-series. Each observed doubly-charged mass is a combination of the entire mass of KLAVNMVPFPR and a fragment from AVFVDLEPTVIDEVR. Calculating the observed doubly-charged mass from sequence information is illustrated for the 942.86 mass. This mass is equal to the neutral mass for KLAVNMVPFPR, [M^0^] = 1270.72 Da; plus the neutral mass for the IDEVR peptide, [M^0^] = 613.33 Da; minus 18 Da for the isopeptide linkage; plus 2 protons; divided by 2. Similar calculations can be made for doubly-charged masses 885, 1042, 1091, 1155, and 1269. Identifying the residues involved in the cross-link is based on the following logic. The only lysine residue in the pair of peptides is Lys-1 from KLAVNMVPFPR, which makes this the lysine component of the isopeptide bond and defines AVFVDLEPTVIDEVR as the peptide containing the acidic component. AVFVDLEPTVIDEVR contains 4 acidic residues that could be the acidic component of the isopeptide bond. Residues Asp-5 and Glu-7 are missing from cross-linked fragments 885 to 1091 *m*/*z* and therefore cannot be part of the isopeptide bond. Fig. 3 shows Asp-12 cross-linked to Lys, but this assignment does not rule out the possibility that the cross-link is to the adjacent Glu.

**Figure 4.**
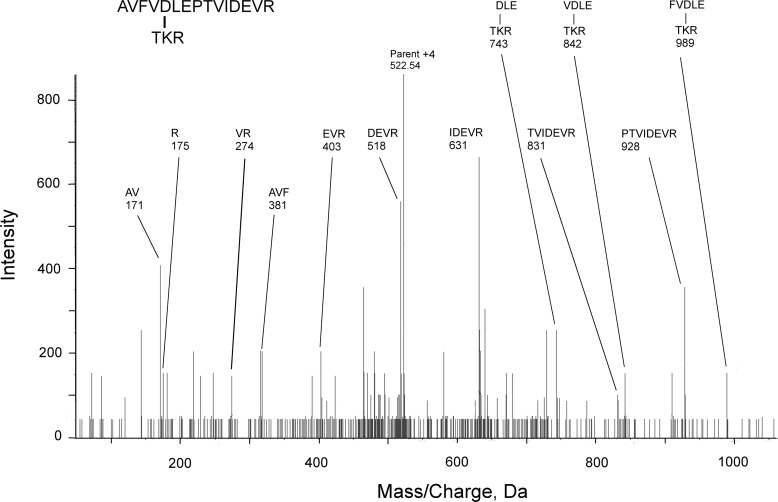
**A mass spectral fragmentation spectrum for the cross-linked peptides AVFVDLEPTVIDEVR from tubulin α1A (P02550) and TKR from tubulin α1A (P02550).** Most unlabeled masses correspond to internal fragments, immonium ions, and *b*- or *y*-ions minus water and/or amine.

The MS/MS spectra for eight of the nine cross-linked peptides in Table 2 contained doubly-charged fragments from which the cross-linked structure was determined. One spectrum contained only singly-charged fragments. Fig. 4 shows the MS/MS spectrum for the cross-linked peptides AVFVDLEPTVIDEVR (α1A, P02550) and TKR (α1A, P02550). As with the spectra containing doubly-charged species, this spectrum consists of three components: 1) the N-terminal *b*-series AVF from AVFVDLEPTVIDEVR; 2) the C-terminal *y*-series PTVIDEVR from AVFVDLEPTVIDEVR; and 3) a singly-charged series that describes the fragmentation of the cross-linked peptide TKR-FVDLE, where the cross-link is between Lys and Asp. Three cross-linked masses are visible. All three contain both an Asp and a Glu residue making either of these residues potential cross-linking partners for the Lys.

All of the lysines involved in these cross-links are also targets for labeling by CPO. All of the lysine partners are either glutamate or aspartate. There is substantial redundancy in the peptides participating in these cross-links. Glutamate 77 or the adjacent aspartate 76, from peptide AVFVDLEPTVIDE^77^VR, appears in five of the cross-linked pairs, and glutamate 113 from peptide ^113^EIIDLVLDR is in 2 pairs. On the other side of the cross-link, lysine 252, from peptide ^252^KLAVNMVPFPR, is part of 3 pairs. Three of the cross-links involve peptides from two different types of tubulin. These cross-links would be expected to promote aggregation. Whether the α-α and β-β cross-links occur between peptides in the same tubulin subunit or between peptides in different subunits is unclear.

### Cross-linked peptides in X-ray structure

**Figure 5.**
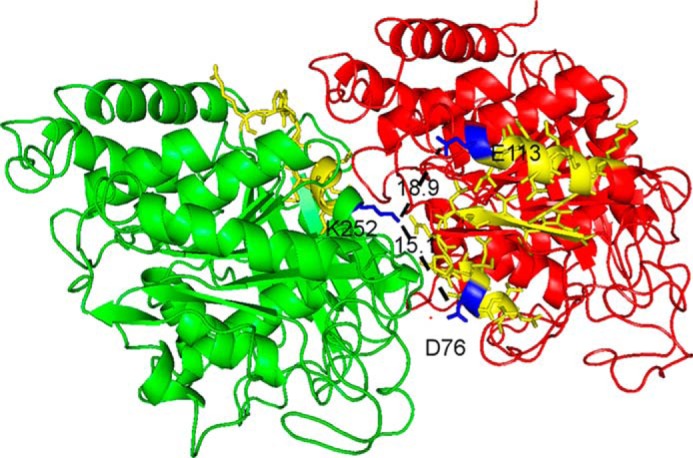
**Crystal structure showing the cross-link between Lys-252 on tubulin β and Asp-76 on tubulin α1A, and the cross-link between Lys-252 on tubulin β and Glu-113 on tubulin α1A.** β-Tubulin is shown in *green*, α1A in *red*. The cross-linked peptides are shown in *yellow*. Lysine 252, aspartate 76, and glutamate 113 are shown in *blue*. The distance between Lys-252 and Asp-76 is 15.1 Å and between Lys-252 and Glu-113 is 18.9 Å, indicated by *dashed lines*.

The cross-linked peptides were located in the X-ray structure of porcine microtubule described by Alushin *et al.* (33) (Protein Data Bank 3J6G). This structure consists of three rows of tubulin α-β dimers with each row containing three α-β dimers for a total of 18 tubulin subunits total. All possible combinations of α-β, α-α, and β-β interactions are represented. Fig. 5 shows a single α-β pair with two intermolecular cross-links, one between Asp-76 from peptide AVFVDLEPTVID^76^EVR and Lys-252 from peptide ^252^KLAVNMVPFPR and another between Glu-113 from peptide ^113^EIIDLVLDR and Lys-252. Both of these cross-links are located at the interface between the α and β subunits.

The distance between Asp-76 and Lys-252 is 15.1 Å. The distance between Glu-113 and Lys-252 is 18.9 Å. Although these distances are long for covalent bond formation, it is worth noting that both KLAVNMVPFPR and AVFVDLEPTVIDEVR are in short α-helices connected via long random coils. EIIDLVLDR is in an α-helix that is connected by two stretches of random coil. All three peptides would be expected to be flexible in solution making it reasonable to suggest that interaction is more likely than the crystal structure would indicate.

Interaction distances for the other cross-linked peptides in Table 2 range from 24 to 54 Å. This observation suggests the microtubule structure in solution is loose enough to allow subunits to move about and interact in ways not apparent from the X-ray structure.

## Discussion

### Mechanism of cross-link formation

Exposure of tubulin to chlorpyrifos oxon results in the modification of the ϵ-amine of lysine or the aromatic hydroxyl of tyrosine. The chemically-activated lysine residues promoted the formation of isopeptide cross-links. This statement is based on the observation that all of the isopeptide cross-linked peptides we have detected were connected through lysines that were also labeled by CPO. Isopeptide bonds were found between lysine and glutamate or aspartate. Cross-links were formed between peptides within a tubulin subunit or between peptides from different tubulin subunits. See Table 2 for details. The observation of CPO-promoted formation of intermolecular cross-links between peptides is new as of this writing. However, Schmidt *et al.* (31) reported that VX promoted intramolecular cyclization within ubiquitin subunits by formation of isopeptide bonds between lysine and glutamic acid.

Intermolecular isopeptide bonds have long been known to stabilize protein structure. Isopeptide bond formation is generally the result of enzyme-mediated reactions between the ϵ-amine from lysine on one protein and either glutamate or glutamine on another. Enzymes involved include transglutaminase (34, 35), sortase (36), the sumoylation complex (37), and the ubiquitin complex (38). By contrast, OP-mediated isopeptide bond formation is not catalyzed by an enzyme. Whether produced by an enzyme or by a chemically-activated lysine, isopeptide bonds are insensitive to reduction (39), resistant to hydrolysis by common proteases (40, 41), and resistant to low voltage collision-induced dissociation in MS (42).

**Figure 6.**
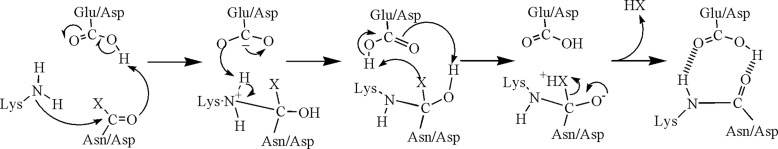
**Autocatalytic isopeptide bond formation mechanism, where *X* is an amine or a hydroxyl (43).**

Intramolecular isopeptide bonds are a more recent discovery (43). They were first reported in 2007 (44) for bacterial cell-surface proteins. They differ from the intermolecular isopeptide bonds in that they form autocatalytically. The proposed mechanism involves a nucleophilic attack by an unprotonated lysine amine on the carbonyl carbon from a nearby asparagine or aspartate. Glutamate or aspartate serves as a proton shuttle (Fig. 6). The reaction occurs in a hydrophobic environment that can alter the p*K_a_* values of the ionizable groups (43).

The finding by Schmidt *et al.* (31) that the organophosphonate VX promotes the formation of intramolecular isopeptide bonds in ubiquitin is another example of an autocatalytic mechanism. Our finding that the organophosphorus toxicant chlorpyrifos oxon promotes intermolecular isopeptide bond formation supports the finding of Schmidt *et al.* (31). In neither study did the untreated protein exhibit isopeptide bonds. This latter observation lends credence to the proposal that OP-labeling is an essential preliminary step in isopeptide bond formation.

**Figure 7.**
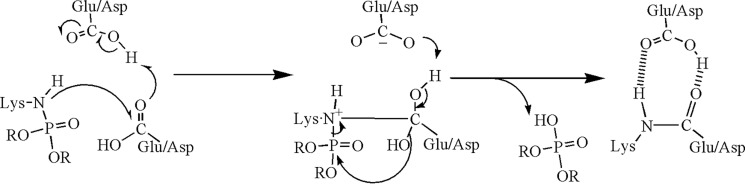
**Organophosphate-promoted isopeptide bond formation between lysine and glutamate or aspartate.** A nearby acidic residue serves as a proton shuttle.

The mechanism of Kang and Baker (43) (Fig. 6) emphasizes the importance of a vicinal acidic group that can shuttle protons and stabilize charged states, thereby promoting the reaction. This concept can be adapted to rationalize OP-promoted isopeptide bond formation. Eight of the 9 cross-linked peptides in Table 2 contain a glutamate or an aspartate close to the cross-linked amino acids. This acidic residue could serve as a proton shuttle similar to the vicinal acidic residue in the Kang mechanism. Because isopeptide bond formation does not occur in the absence of OP it clearly implicates OP in the mechanism of isopeptide bond formation. It is tempting to suggest that OP promotes isopeptide bond formation because the phosphonyl moiety is a better leaving group than hydroxyl or amine in the Kang mechanism. A tentative mechanism is shown in Fig. 7.

The hydroxyl group on tyrosine can also be covalently modified by CPO. Using LC-MS/MS and the Batch Tag search method we have identified a few cross-linked peptides involving diethoxyphosphotyrosine. These will be described in a separate report.^4^

### Protein aggregation induced by OP

The formation of cross-links between tubulin subunits caused tubulin to aggregate. The extent of aggregation depended on the concentration of CPO used to treat the tubulin. Aggregation was most extensive at 1.5 mm CPO. However, even at 1.5 μm CPO cross-linked trimers were apparent (Fig. 2). The presence of tubulin cross-links explains the CPO-induced high-molecular-weight aggregates observed on SDS-PAGE in Fig. 1. Although the present study focused on tubulin, we expect that cross-linking *in vivo* is likely to occur between nonidentical proteins, for example, between tubulin and microtubule-associated proteins.

The 1.5 mm CPO concentration used for most of our experiments is high. Low level exposure in humans is expected to correspond to about 25 nm CPO, a dose that would inhibit 50% of the plasma butyrylcholinesterase, but would not be accompanied by toxic symptoms or AChE inhibition. Humans poisoned with chlorpyrifos had up to 12 μm CPO in blood at the time of hospital admission 5 h post-ingestion (45). The cross-linking observed at 1.5 mm CPO in our experiments is far more extensive than could be expected from low dose exposures. The primary purpose of our experiments was to demonstrate that CPO-labeled lysine could promote cross-linking. We labeled with a high level of CPO to ensure that CPO-labeled lysine was formed. Once diethoxyphospholysine has been formed the cross-linking reactions are independent of the CPO concentration. Our experiments show that CPO adducts on lysine are capable of cross-linking proteins. This finding is a proof of that principle. We also showed that 1.5 μm CPO was capable of forming cross-linked trimers. We hypothesize that exposure to lower doses will form only a few cross-links, and that the cross-linked proteins could accumulate as misfolded proteins and lead to neurotoxicity.

### Medical relevance of tubulin modification, implications for neurotoxicity

Both simple modification of tubulin by OP and tubulin aggregation would be expected to disrupt the structure and function of tubulin in neurons. This in turn could lead to the neurodevelopmental problems observed in children and the chronic neurotoxicity observed in adults who have been exposed to OP. Tubulin is only an example of proteins that could become aggregated when exposed to OP. OP-mediated cross-linking and aggregation of other proteins is likely to occur.

OP-induced protein aggregation fits well into the Brady hypothesis (46, 47) for neurodegenerative diseases. Brady and co-workers (46, 47) have developed a unifying hypothesis to explain neurodegenerative diseases including Alzheimer's, Parkinson's, Huntington's, and prion diseases as follows. 1) Aggregated proteins stimulate the activity of kinases. 2) The activated kinases phosphorylate motor proteins in excess. 3) The excessively phosphorylated motor proteins drop their cargo. 4) Loss of cargo deprives neurons of constituents that maintain neuron function. 5) Neurons slowly lose their connections to other neurons, which results in 6) clinical symptoms. The Brady hypothesis proposes that specific kinase networks and specific neurons are involved in each disease, but that the overall sequence of events is shared. Aggregated proteins induce excessive phosphorylation of proteins involved in axonal transport, which in turn disrupts fast axonal transport and causes neurotoxicity. We hypothesize that chronic neurotoxicity from low level CPO exposure fits the Brady model, with the difference being that “step 0” is OP-adduct formation.

Despite the proposed connection between the Brady hypothesis and our tubulin results, the correlation is highly speculative. Neurological sequelae due to exposure to CPO might be the consequence of CPO modification/aggregation of other proteins. It is also important to note that all protein aggregates are not the same (48). There are three major types of protein aggregates: amyloid fibrils, amorphous deposits, and native-like deposits. Each is associated with different types of disease. Amorphous deposits and amyloid fibrils are assemblages of small peptides. Native-like deposits derive from fully-folded proteins (48). The tubulin aggregates described in the current work would be most closely related to the native-like deposits. None of the protein aggregates associated with neurodegenerative disorders are believed to require covalent cross-linking for initiation (48).

The published literature on the effects of OP treatment fits the Brady hypothesis by supporting a link between OP exposure, hyperphosphorylation of proteins involved in axonal transport, impaired axonal transport, and chronic illness. Hyperphosphorylation following OP treatment has been reported for neurofilaments (49), Tau protein (50), calcium/cAMP-response element-binding protein (13), tubulin and microtubule-associated protein 2 (51). Studies in cell-free systems and in animals provide evidence for impaired axonal transport and decreased neuron synaptic spine density as consequences of treatment with OP (14, 52, 53). Our results from the current work suggest that chronic neurotoxicity from OP exposure could be initiated by OP-adduct formation followed by isopeptide bond formation and protein aggregation.

## Materials and methods

### CPO labeling and trypsin digestion of tubulin

One milligram of porcine tubulin (lyophilized powder >99% pure; Cytoskeleton, Denver, CO, catalog number T240) was dissolved in 3.6 ml of 20 mm Tris/Cl buffer, pH 8.5, containing 0.01% sodium azide to give 0.275 mg of tubulin/ml. This was mixed with 2.5 μl of 0.3 m chlorpyrifos oxon (Chem Service, West Chester, PA, catalog number MET-11459B) in ethanol to make 1.5 mm CPO. The reaction was incubated for 8 days at 24 °C. Tubulin remained in solution. Excess CPO was separated from 50 μl of protein by size exclusion chromatography on a Prob Quant G50 micro-column (GE Healthcare catalog number 28-9034-08) while simultaneously changing the buffer to 20 mm ammonium bicarbonate, pH 8. CPO-tubulin in 20 mm ammonium bicarbonate was digested with 0.5 μl of porcine trypsin (0.4 μg/μl, sequencing grade modified; Promega, Madison, WI, catalog number V511C) at 37 °C overnight. The digest was dried and resuspended in 20 μl of 0.1% formic acid in preparation for MS. The digests were stored at −20 °C. Unlabeled, control porcine tubulin was treated similarly and analyzed by LC-MS/MS for diethylphosphate labeling and cross-linked peptides.

### Isopeptide hydrolysis

Isopeptide bonds can be selectively hydrolyzed by γ-glutamyltranspeptidase (horse kidney; Sigma, catalog number G9270) (54). Hydrolysis was conducted as described (39). One microgram of CPO-tubulin or untreated porcine tubulin was incubated with 1 unit of γ-glutamyltranspeptidase and 10 mm Gly–Gly peptide (Sigma, catalog number G1002) in 65 μl of 50 mm Tris/Cl, pH 8.0, containing 150 mm sodium chloride at 37 °C for 22 h.

### SDS-PAGE

A 4–20% gradient Mini Protean TGX Stain-free 10-well, 0.75-mm thick polyacrylamide precast gel was used (Bio-Rad, catalog number 456-8094) in a Mini Protean Tetra PAGE cell (Bio-Rad, catalog number 1658004). Tank buffer was 60 mm Tris/Cl, pH 8.1. The top buffer was 25 mm Tris glycine, pH 8.4, containing 0.1% SDS. Gels used for Western blotting were loaded with 1 μg of tubulin/well. Gels used for Coomassie staining were loaded with 5 μg of tubulin/well. Before loading, tubulin was mixed in a 1 to 3 ratio with 93.8 mm Tris/Cl, pH 6.8, containing 15% glycerol, 3% SDS, 0.3 m DTT, and 0.0018% bromphenol blue, and heated in a boiling water bath for 3 min. Electrophoresis at room temperature and 150 volts was stopped when the bromphenol blue began to come off the bottom of the gel (1.5 h). Coomassie staining employed 0.025% Coomassie Blue in 50% methanol, 10% acetic acid for about 2 h, followed by destaining in water.

### SDS-PAGE Western blotting

A Mini Protean II Western blotting cell (Bio-Rad) was used to transfer the proteins from SDS-PAGE to Immuno-Blot PVDF membrane (Bio-Rad, catalog number 162-0177). The transfer sandwich was assembled according to the manufacturer's instructions. Transfer was performed in 25 mm Tris glycine buffer, pH 8.4, using 70 volts at 6 °C, for 1 h. The membrane was blocked with TBS/Tween (TBST, 20 mm Tris/Cl, pH 7.4, plus 150 mm sodium chloride, 0.05% Tween 20) containing 5% nonfat dry milk (Carnation). The membrane was hybridized with a mouse mAb, 0.27 μg/ml of depY, which targeted diethoxyphosphotyrosine (55) or with a sheep polyclonal that targeted tubulin (Cytoskeleton, catalog number ATN02) in TBST plus 5% nonfat dry milk, for 18 h at 6 °C. After washing with TBST, the membrane was hybridized with a 1 to 5000 dilution of horse anti-mouse IgG conjugated to horseradish peroxidase (Cell Signaling, Danvers, MA, catalog number 7076) or with a 1 to 5000 dilution of donkey anti-sheep IgG conjugated to horseradish peroxidase (The Binding Site Limited, Birmingham, UK, catalog number PP360) in TBS plus 5% nonfat dry milk, for 1.5 h at room temperature.

The labeled proteins were visualized with Super Signal West Dura Extended Duration fluorescent substrate for horseradish peroxidase (Thermo Scientific, Rockford, IL, catalog number 27071). Chemiluminescence was detected with an Azure Biosystems model C600 flat plate scanner (Azure Biosystems, Dublin, CA).

### Capillary electrophoresis Western blotting

Simple Western capillary electrophoresis was performed on tubulin and CPO-treated tubulin by RayBiotech, Inc. (Norcross, GA). In one analysis, the primary antibody was a mouse monoclonal IgG1κ anti-isopeptide antibody 81D1C2 (Abcam, Cambridge, MA, catalog number ab422). In another analysis, the primary antibody was sheep polyclonal anti-α/β-tubulin (Cytoskeleton, catalog number ATN02). One microliter of tubulin, at 0.1 mg/ml, was loaded onto the capillary electrophoresis device. After electrophoresis, proteins were immobilized to the capillary wall via a proprietary, photoactivated capture chemistry. Matrix was removed and the primary antibody at a concentration of 0.02 mg/ml was passed through the capillary. Antibody-bound proteins were located using an HRP-conjugated secondary antibody together with a chemiluminescent substrate (ProteinSimple PS-CS01).

### Mass spectral data acquisition

Electrospray ionization MS was performed on an AB Sciex 6600 Triple TOF instrument (AB Sciex, Framingham, MA). The peptide preparation (about 1 μg/μl) was acidified with 0.1% formic acid. Five microliters were subjected to HPLC separation using a cHiPLC Nanoflex microchip column (Eksigent, Dublin CA) attached to a splitless Ultra 1D Plus ultra-high pressure chromatography system (Eksigent, Dublin, CA). The Nanoflex microchip system consisted of a replaceable microfluidic trap column (200 μm × 0.5 mm) and separation column (75 μm × 15 cm separation) both packed with ChromXP C18 resin (3 μm, 120 Å particles). Solvents were water/acetonitrile/formic acid (A: 100/0/0.1%; B: 0/100/0.1%). The sample was loaded onto the trap column and washed for 15 min at 2 μl/min with Solvent A to remove salts. The flow rate was reduced to 0.3 μl/min, and the sample was eluted using a 65-min linear gradient ranging from 95% solvent A, 5% solvent B to 70% solvent A, 30% solvent B. Effluent from the HPLC column was sprayed directly into the mass spectrometer. Mass spectra were collected in positive mode, over a mass range of 200 to 3000 Da, using an accumulation time of 250 ms, a collision energy of 10 V, a declustering potential of 70 V, an ion spray potential of 2700 V, and an interface heater temperature of 150 °C. Peptide fragmentation was accomplished by collision-induced dissociation using nitrogen as the collision gas at a pressure of 2 × 10^−5^ torr. A maximum of 50 parent ions were subjected to fragmentation per cycle. Fragmentation spectra were collected in positive mode, over a mass range of 50–3000 Da, using an accumulation time of 25 ms, a collision energy determined by the software (rolling), and a collision energy spread of ±10 V. Peptides to be fragmented were chosen by an information directed acquisition algorithm using charge states 1 to 4, minimum signal of 100 cps, with no parent ion exclusion.

### Protein Pilot analysis of the mass spectral data

Data acquired by the Triple TOF mass spectrometer (*.wiff files) were searched against a Uniprot-Sprot protein database using the Paragon algorithm from Protein Pilot software version 5.0 (AB Sciex). Search parameters included Cys alkylation = none, digestion = trypsin, species = *Sus scrofa*, ID focus = biological modifications, database = unipro_sprotJan2015.fasta, search effort = thorough ID, and false discovery rate = 0.05. In-house modifications to the Special Factors section included modification of serine, tyrosine, threonine, lysine, histidine, and cysteine by diethoxyphosphate and monoethoxyphosphate. Protein Pilot identified the tubulin peptides including those that were candidates for organophosphate modification, but did not report cross-linked peptide candidates.

### Batch Tag (web) analysis of the mass spectral data for cross-linked peptides

The *.wiff mass spectral data included information on cross-linked peptides. The data could be accessed by the Batch Tag (web)/Search Compare algorithms from ProteinProspector (UCSF Mass Spectrometry Facility) as follows. Protein Pilot *.wiff files were converted to a *.mgf file format for use by Batch Tag (web) using Protein Pilot menu item File/Export/MGF Peaklist (56, 57). Batch Tag (web) searches were configured to identify pairs of peptides that were cross-linked via isopeptide bonds. Critical parameters for Batch Tag were chosen based in large part on the recommendations of Trnka *et al.* (56). Parameters used for all analyses included database = Swissprot.2016.9.6.random.concat, taxonomy = *S. scrofa*, digest = trypsin, constant modifications = none; protein *M*_r_ = 1000 to 70,000, accession number = P02550 (for tubulin α1A), P02554 (for tubulin β), Q767L7 (for tubulin β), and Q2XVP4 (for tubulin α1B), parent Tol = 20 ppm, fragment Tol = 30 ppm, and variable mods = oxidation (M), uncleaved = unchecked, link search type = user defined link. Other parameters were adjusted to match the type of cross-link being searched. For cross-links between lysine and glutamate, aspartate, glutamine, or asparagine: AA table = Asp, Glu, Gln, Asn, and Lys checked, Link AAs = Glu, Asp, Protein C-term → Lys, Protein N-term, and Bridge Elem Comp = H-2O-1. For cross-links between lysine and any amino acid: AA table = all checked, link AAs = Lys, Protein N-term → Ala, Cys, Asp, Glu, Phe, Gly, His, Ile, Lys, Leu, Met, Asn, Pro, Gln, Arg, Ser, Thr, Val, Trp, Tyr, and Bridge Elem Comp = H-2O-1. Search Compare parameters included: accession numbers P02550 (for tubulin α1A), P02554 (for tubulin β), Q767L7 (for tubulin β), and Q2XVP4 (for tubulin α1B), *m*/*z*, charge, score difference, coverage, DB peptide, menu = mods in peptide, links, time, cross-linked peptide, report type = cross-linked peptides, and sort type = start residue.

The identity of each cross-linked candidate found by Batch Tag/Search Compare, regardless of the Score or Score Difference value, was checked by manual sequencing using Peak View software (version 1.2, AB Sciex). Peak View reads all of the data in the *.wiff file, including that which is not reported by Protein Pilot. The cross-linked candidate was located based on its observed monoisotopic mass, charge state, and elution time, all of which were obtained from the Search Compare report. Multiple occurrences of a given candidate were summed before the fragmentation spectrum was analyzed.

Identification of a cross-linked peptide is based on the presence of two peptides in the fragmentation spectrum. Each peptide pair that we report exhibited sequence information from both peptides and masses that corresponded to cross-linked portions from both peptides. The cross-linked masses are typically of low abundance but they provide a means for identifying the specific amino acids involved in the cross-link (56, 58). Most of the cross-links were identified by analyzing multiply-charged fragment ions as described by Liu *et al.* (59).

## Author contributions

L. M. S. resources; L. M. S. and O. L. data curation; L. M. S. and O. L. formal analysis; L. M. S. validation; L. M. S. and O. L. investigation; L. M. S. and O. L. methodology; L. M. S. and O. L. writing-original draft; L. M. S. and O. L. writing-review and editing; O. L. conceptualization; O. L. funding acquisition; O. L. project administration.

## Footnotes

4 L. M. Schopfer and O. Lockridge, unpublished data.

## Footnotes

3 The abbreviations used are:

AChE: acetylcholinesterase
CPO: chlorpyrifos oxon
CPS: chlorpyrifos
OP: organophosphorus toxicants
PVDF: polyvinylidine fluoride
depY: anti-diethoxyphosphotyrosine antibody
VX: *O*-ethyl methyl S-2-(diisopropylaminoethyl)phosphonothioate).
